# Heritability and genetic associations of triglyceride and HDL-C levels using pedigree-based and empirical kinships

**DOI:** 10.1186/s12919-018-0133-x

**Published:** 2018-09-17

**Authors:** Nicholas B. Blackburn, Arthur Porto, Juan M. Peralta, John Blangero

**Affiliations:** 1 South Texas Diabetes and Obesity Institute, Department of Human Genetics, University of Texas Rio Grande Valley School of Medicine, One University Blvd., Modular Building #100, Brownsville, TX 78250 USA; 2 Menzies Institute for Medical Research, University of Tasmania, Liverpool St, Hobart, TAS 17 Australia

## Abstract

The heritability of a phenotype is an estimation of the percent of variance in that phenotype that is attributable to additive genetic factors. Heritability is optimally estimated in family-based sample populations. Traditionally, this involves use of a pedigree-based kinship coefficient generated from the collected genealogical relationships between family members. An alternative, when dense genotype data are available, is to directly measure the empirical kinship between samples. This study compares the use of pedigree and empirical kinships in the GAW20 data set. Two phenotypes were assessed: triglyceride levels and high-density lipoprotein cholesterol (HDL-C) levels pre- and postintervention with the cholesterol-reducing drug fenofibrate. Using SOLAR (Sequential Oligogenic Linkage Analysis Routines), pedigree-based kinships and empirically calculated kinships (using IBDLD and LDAK) were used to calculate phenotype heritability. In addition, a genome-wide association study was conducted using each kinship model for each phenotype to identify genetic variants significantly associated with phenotypic variation. The variant rs247617 was significantly associated with HDL-C levels both pre- and post-fenofibrate intervention. Overall, the phenotype heritabilities calculated using pedigree based kinships or either of the empirical kinships generated using IBDLD or LDAK were comparable. Phenotype heritabilities estimated from empirical kinships generated using IBDLD were closest to the pedigree-based estimations. Given that there was not an appreciable amount of unknown relatedness between the pedigrees in this data set, a large increase in heritability in using empirical kinship was not expected, and our calculations support this. Importantly, these results demonstrate that when sufficient genotypic data are available, empirical kinship estimation is a practical alternative to using pedigree-based kinships.

## Background

SOLAR (Sequential Oligogenic Linkage Analysis Routines) [1], software developed for the genetic analysis of pedigrees, can be used to calculate the heritability (h^2^) of a phenotype. This calculation requires the phenotype measurement, relevant covariates, and a kinship matrix. Traditionally, the kinship matrix is derived from a carefully curated pedigree (or pedigrees) joining together the individuals with phenotypes by their self-reported genealogical relationships. The use of self-reported genealogical relationships has one obvious drawback: incorrectly specified relationships. These pedigree errors can arise for multiple reasons, including paternity, recording errors, as well as cultural differences in the understanding of the definition of biological kinship relationships. In addition, when a cohort of pedigrees is recruited from the same geographical region, it’s possible that there may be unknown kinship connections between seemingly discrete pedigrees.

Accurate biological relationships are necessary for the calculation of phenotype heritability. Uncertainty surrounding pedigree relationships in a data set reduces the power of heritability calculations and leads to inaccurate results at best, or false results at worst.

With the availability of dense genotyping array data, a potential solution to this problem is to employ the use of empirical kinship estimates. Empirical kinship is when the kinship between each individual in a cohort is estimated using dense genotyping data from single-nucleotide polymorphism (SNP) arrays or next-generation sequencing. Empirical kinship estimates will overall closely align with the kinship calculated from pedigrees, but, importantly, are also able to clarify pedigree relationships, provide an additional quality-control measure to identify sample swaps or duplicates, identify unknown or distant relationships, and overall remove the need to rely on genealogical records. Furthermore, where individuals are unrelated in a pedigree kinship matrix, some level of empirical kinship can be calculated for all pairs in the data set.

Intuitively, the use of a matrix of empirical kinship estimates should improve heritability calculations as the observed kinship measurement is used rather than the kinship expectation based on genealogy. We examined in the GAW20 data set from the Genetics of Lipid Lowering Drugs and Diet Network (GOLDN) study [2] how employing empirical kinship specifically affects heritability calculations. We used SOLAR for all heritability calculations and for the calculation of the pedigree kinship matrix using the pedigrees provided in the GAW20 data set. To calculate the empirical kinship matrices we used 2 established methods: LDAK [3] and IBDLD [4]. We further extended this analysis by using measured genotype-association testing in SOLAR to identify variants that are associated with the phenotypes under examination. We hypothesize that using empirical kinships will strengthen the association results and effect sizes detected in comparison to the use of pedigree kinships.

## Methods

### Data set

The distributed GAW20 genotypes of 718,544 autosomal SNPs were converted to their corresponding DNA nucleotide bases and the hg18 mapping coordinates were uplifted to hg19. This resulted in 718,407 SNPs for analysis, with 135 excluded because of failing the conversion to hg19. The pedigree distributed with the GAW20 data set was converted to SOLAR format. The phenotype data distributed with the GAW20 data set was merged into a single SOLAR format phenotype file.

### Prest-plus analysis within-pedigrees and across-pedigrees

Prest-Plus [5] was used to assess recorded pedigree relationships and to identify evidence of relatedness outside of the GAW20 pedigrees. Using PLINK (v1.90b3m) [6], GAW20 genotypes were linkage disequilibrium pruned (−-indep-pairwise 2000 10 0.1) and Hardy-Weinberg equilibrium pruned (nominal significance of *P* = 0.05 used as the threshold) resulting in 22,697 SNPs for within-pedigree and across-pedigree Prest-Plus analysis.

### Empirical kinship calculation

**Fig. 1:**
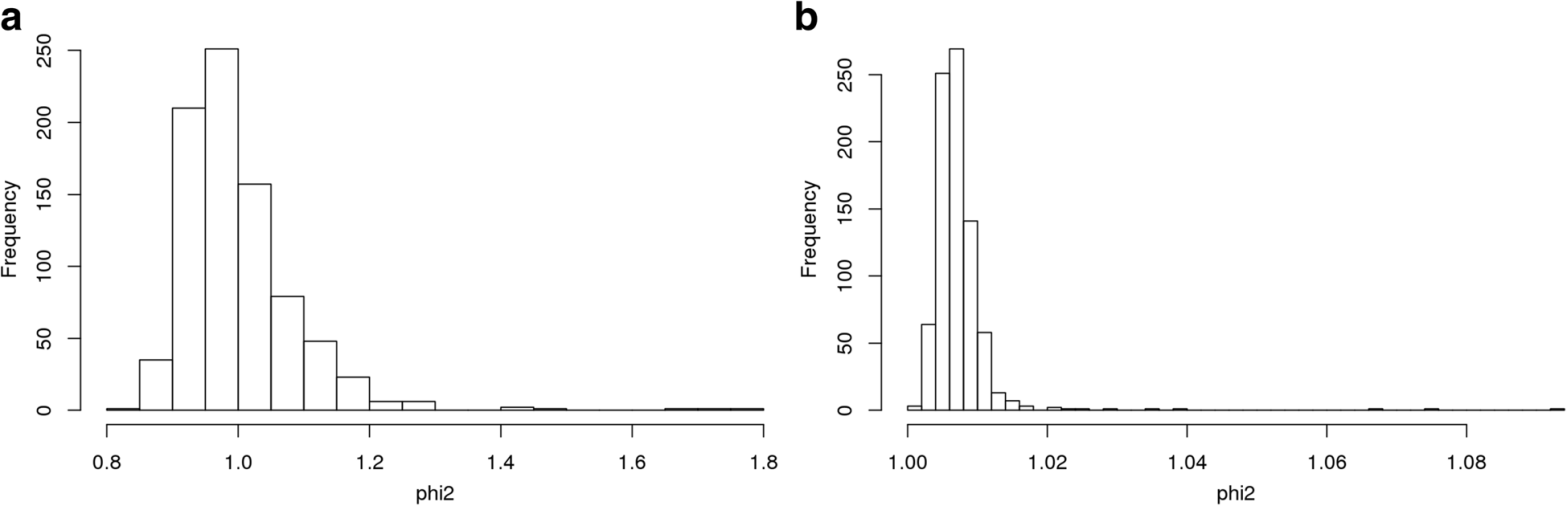
Distribution of diagonal entries (estimates of self-relatedness) in the unscaled matrices from (**a**) LDAK and (**b**) IBDLD

LDAK version 4.9 [3] and IBDLD version 3.33 [4] were used to derive 2 empirical kinship matrices based on the GAW20 genotype data. For LDAK, in principle, this kernel should correspond to a genetic relationship matrix; in practice, however, we observed that LDAK estimates of self-relatedness were widely spread around their expectation of 1 (Fig. 1a). For IBDLD the estimates of self-relatedness were closer to 1 (Fig. 1b). The empirical kinship estimate matrices from LDAK and IBDLD were postprocessed to remove negative nonzero values and scaled to have a diagonal equal to 1.

### SOLAR heritability analysis

The 2 phenotypes assessed were triglyceride levels and high-density lipoprotein cholesterol (HDL-C) levels pre- and post-fenofibrate intervention. For an individual, when multiple phenotype measurements were available at the 2 visits pre- or 2 visits post-fenofibrate intervention, these were averaged into single pre- and postintervention phenotype values; otherwise, the single pre- or postmeasurement was used. Phenotypes were analyzed using SOLAR (SOLAR Eclipse version 7.6.4) [1]. All phenotypes were residualized with SOLAR for the available covariates, including age, sex, their interactions (age × sex, age^2^, age^2^ × sex), study center, smoking, and principal components 1 to 4 (to control for possible population stratification, estimated only on pedigree founders using the SNP data in R and projected to the full sample set). Residualized phenotypes were inverse-normalized in SOLAR to prevent nonnormal distribution errors during analysis, ensuring that all phenotypes had a mean of 0 and SD of unity. Heritability was estimated using SOLAR’s variance components framework. These analyses were completed separately using the pedigree kinship matrix derived from SOLAR and each of the empirical kinship matrices.

### Measured genotype analysis

Single-variant association testing was conducted using measured genotype analysis (MGA) in SOLAR for the 718,407 SNPs available for analysis in the GAW20 data set. This analysis takes into account the nonindependence of participants, using the kinship matrix, incorporating each SNP separately into the analysis model as a covariate measured as a genotype dosage (0, 1, 2) and evaluating the genotype-specific difference in the phenotype means. For genome-wide suggestive significance a *P*-value threshold of *P* ≤ 1.00 × 10^− 5^ was used, and for Bonferroni-corrected genome-wide significance a threshold of *P* ≤ 6.9 × 10^− 8^ was applied. Manhattan plots of MGA results were constructed in R using qqman [7].

## Results

### Within-pedigree relationship analysis and detection of distant relationships between unrelated samples

**Fig. 2:**
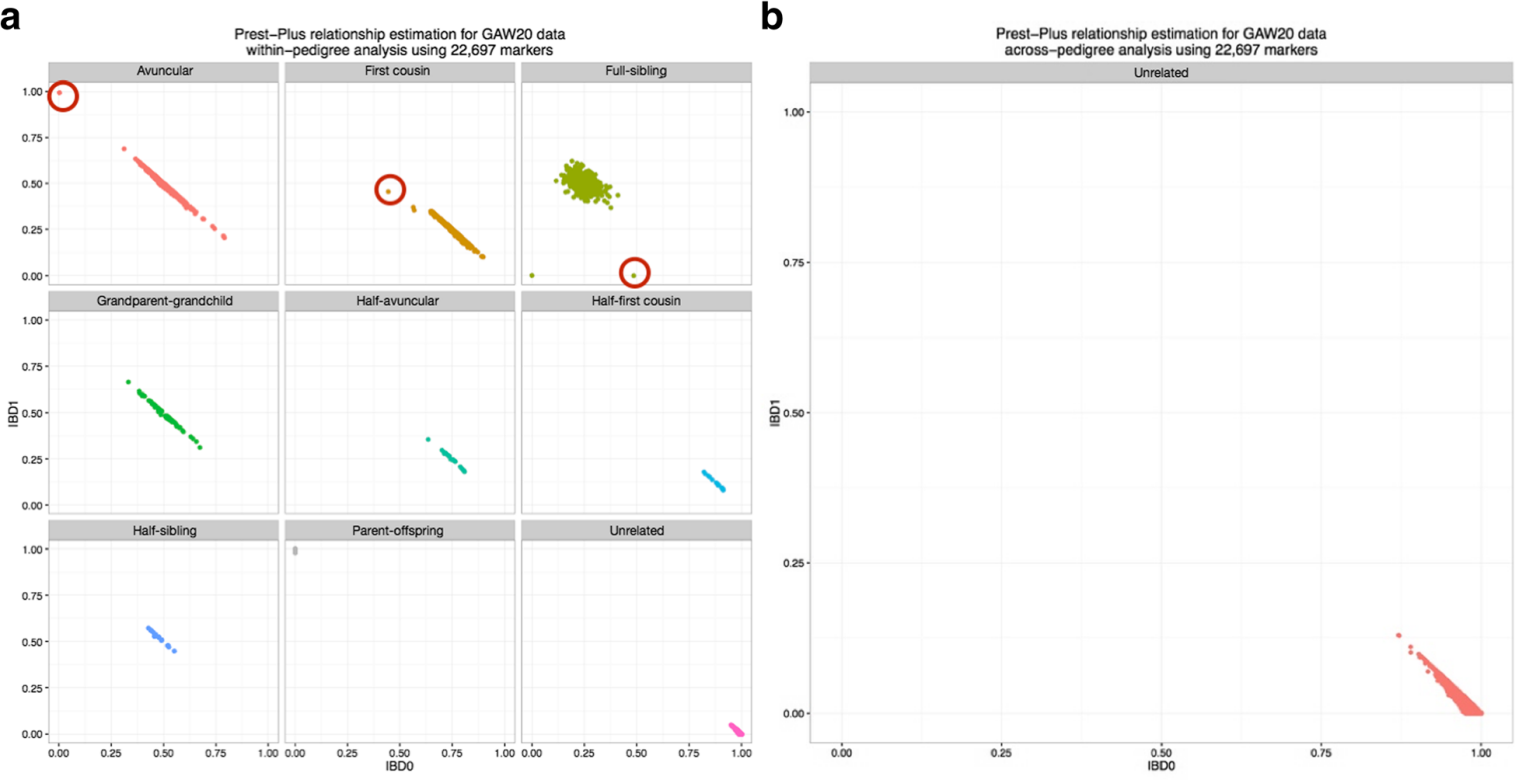
Prest-Plus relationship analysis of the GAW20 data set from the GOLDN study. (**a**) Within-pedigree analysis; monozygotic twins are included in the full-sibling subset, and (**b**) across-pedigree relationship analysis

Prest-Plus identified unexpected relationships within the GAW20 data set when assessing relationships within-pedigrees (Fig. 2a), and limited evidence of distant relationships outside of the pedigree between “unrelated” individuals (Fig. 2b). The unexpected relationships based on the within-pedigree analysis suggest sample swap issues and the samples contributing to these errors were excluded from the data set (samples circled in Fig. 2a and summarized in Table 1).

**Table 1:** Erroneous samples identified through Prest-Plus within-pedigree analysis

Family ID	Individual IDs	Expected relationship	Measured relationship
198	5604, 8117	Avuncular	Parent–offspring
375	1927, 4078	Full-sibling	Unknown
198	3621, 8117	First cousin	Full-sibling

### Heritability of triglyceride and HDL-C levels pre- and post-fenofibrate intervention, using SOLAR with pedigree-based and empirical kinship

Heritability estimates using SOLAR identified that both triglyceride levels and HDL-C were significantly and highly heritable pre- and post-fenofibrate intervention (Table 2), regardless of whether IBDLD, LDAK, or pedigree kinship was used. General observations that can be made are that LDAK consistently estimated the lowest heritability of the 3 methods with pedigree-based and IBDLD-based estimates comparably similar. A decrease in sample size for triglyceride post-fenofibrate intervention, which is a factor of the samples measured and genotyped in the GOLDN data set, correspondingly decreases the magnitude of heritability estimates for the phenotype, except for estimates using LDAK.

**Table 2:** Heritability estimates of triglyceride and HDL-C phenotypes using pedigree-based and empirical kinships

Phenotype	Kinship	h^2^	*p* Value	h^2^ SE	Sample size
Triglyceride pre-fenofibrate	Pedigree	0.424	6.09E-11	0.076	817
	IBDLD	0.443	4.71E-11	0.075	817
	LDAK	0.335	8.79E-10	0.064	817
Triglyceride post-fenofibrate	Pedigree	0.397	1.59E-09	0.078	774
	IBDLD	0.404	3.75E-09	0.078	774
	LDAK	0.350	4.20E-10	0.065	774
HDL-C pre-fenofibrate	Pedigree	0.553	4.05E-20	0.068	817
	IBDLD	0.545	1.17E-19	0.065	817
	LDAK	0.480	2.02E-18	0.059	817
HDL-C post-fenofibrate	Pedigree	0.580	8.82E-21	0.068	817
	IBDLD	0.561	6.79E-20	0.064	817
	LDAK	0.472	9.38E-17	0.061	817

### Measured genotype association analysis using SOLAR of triglyceride and HDL-C measurements, using both pedigree-based and empirical kinship

**Fig. 3:**
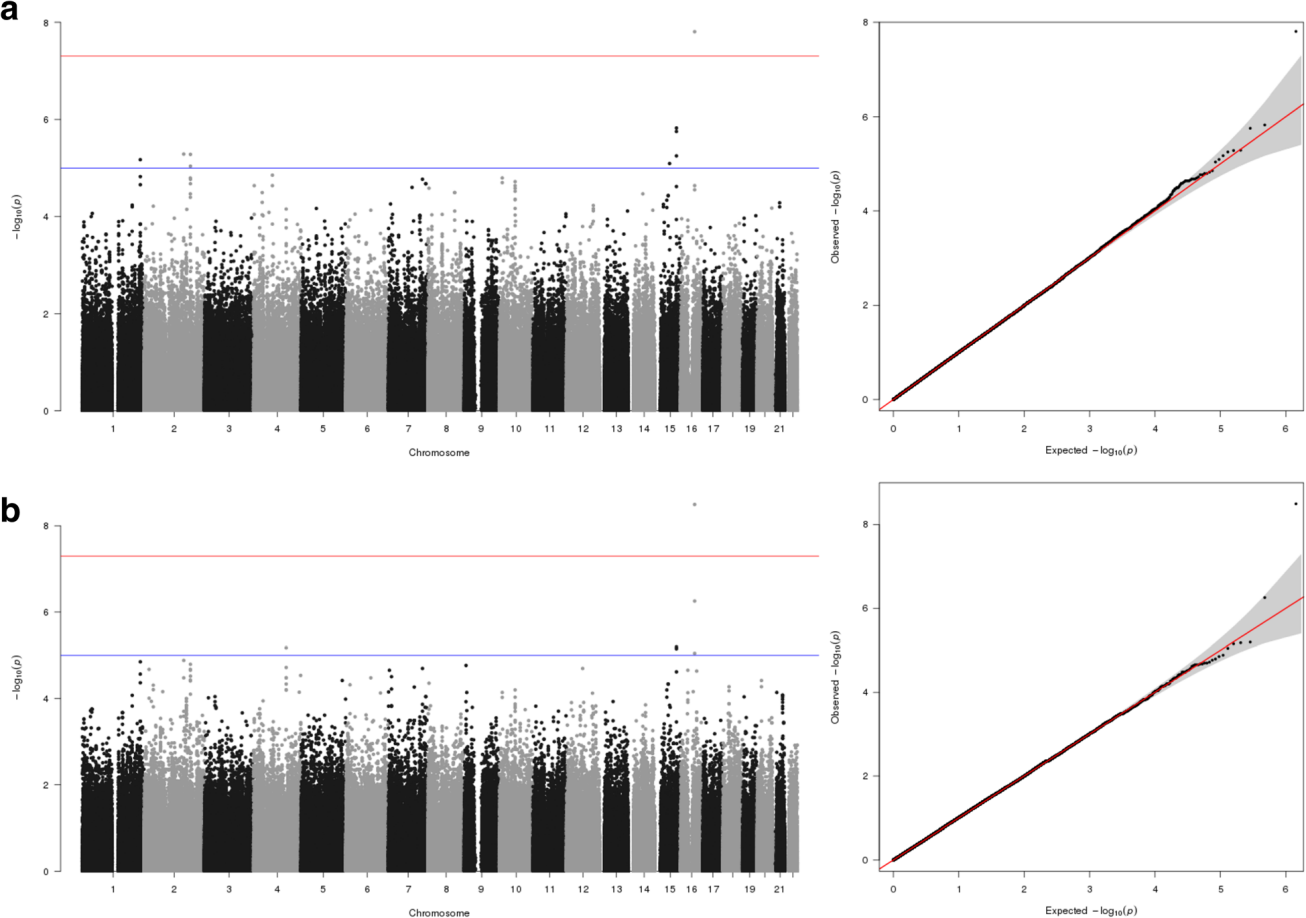
Manhattan and Q-Q plots of results from MGA analysis using pedigree kinships in SOLAR for (**a**) HDL-C pre-fenofibrate intervention and (**b**) post-fenofibrate intervention

MGA of 718,407 SNPs across both triglyceride and HDL-C, pre- and post-fenofibrate intervention identified 1 genome-wide significant SNP, rs247617 on chromosome 16, associated with HDL-C pre- and post-fenofibrate intervention under all 3 kinship models. Figure 3 shows the Manhattan and quantile–quantile (Q-Q) plots for the MGA results of HDL-C measurements for the pre- and post-fenofibrate interventions for pedigree-based kinship (IBDLD and LDAK results not shown). Table 3 summarizes the association results for rs247617. Even though the data are not shown here, associations with suggestive significance were observed for triglyceride levels both pre- and post-fenofibrate intervention. Indeed, in the companion paper by Peralta et al. in which a genome-wide linkage analysis of the triglyceride levels from the GAW20 GOLDN data set was conducted, a linkage peak was detected on chromosome 10, covering the region of the strongest MGA association for that phenotype in this study [8].

**Table 3:** MGA identifies SNP rs247617 associated with HDL-C levels

Phenotype	Kinship model	Chi	p.SNP	Beta SNP	Beta SNP (SE)
HDL-C pre-fenofibrate	Pedigree	31.97	1.56 × 10^−8^	0.314	0.056
	IBDLD	32.27	1.34 × 10^−8^	0.315	0.055
	LDAK	32.55	1.16 × 10^−8^	0.309	0.054
HDL-C post-fenofibrate	Pedigree	35.07	3.18 × 10^−9^	0.329	0.055
	IBDLD	35.97	2.00 × 10^−9^	0.332	0.055
	LDAK	35.60	2.43 × 10^−9^	0.324	0.054

## Discussion

The analysis presented here using the GAW20 data set from the GOLDN study sought to examine whether the use of empirical kinship for the estimation of phenotype heritability and genetic associations in a data set of related individuals was an improvement over relying on pedigree-based kinship. From this analysis, we determined that empirical kinship is analogous, if not equivalent, to pedigree-based kinship. A limitation of the current data set was the minimal unknown relatedness outside of the known pedigrees. It could be expected that in a data set with greater unknown relatedness, or incorrect relatedness (eg, full-siblings reported, when empirically the pair are half-siblings) that heritability estimations from pedigree-based and empirical kinships would be more divergent, with the empirical more accurate.

Pedigree-based kinship in this data set resulted in the highest heritability estimates, with empirical kinships from LDAK generating the lowest heritability estimates. IBDLD empirical kinship resulted in heritability estimates most similar to the pedigree-based estimates. Both phenotypes used from this data set, triglyceride and HDL-C measurements, were significantly heritable pre- and post-fenofibrate intervention, indicating a strong genetic component to phenotype variation.

MGA in SOLAR, accounting for the nonindependence of related samples, identified 1 genome-wide significant SNP, rs247617, associated with HDL-C levels (see Fig. 3). rs247617 has previously shown evidence of association with HDL-C levels [9], low-density lipoprotein (LDL) levels [10] and metabolic syndrome [11]. rs247617 is located upstream of the gene *CETP* (cholesteryl ester transfer protein). The protein product of *CETP* is found in the plasma and has the role of transferring cholesterol esters from HDL-C to LDL [12]. Defects in *CETP* are reported to be the cause of hyperalphalipoproteinemia 1 (HALP1), a disease characterized by abnormally elevated levels of HDL-C [13, 14]. Genetic associations of suggestive genome-wide significance, not reported here, were observed in a linkage peak identified in the companion paper by Peralta et al. [8]. Furthermore, the companion paper by Porto et al. shows that genetic association studies can benefit from the use of empirical genetic values in the context of genomic predictions [15]. Using the empirical genetic values calculated for triglyceride and HDL-C may identify additional genome-wide significant associations.

To further examine the strength of using empirical kinship, the known pedigrees in this data set could be selectively broken into smaller pedigrees, to reduce the pedigree kinship matrix. We could then assess whether the triglyceride and HDL-C phenotypes remain significantly heritable, whether genetic associations detected using the full pedigree kinship matrix are replicated and whether in this context whether stronger support is provided for using empirical kinship in phenotype heritability estimation and genetic association studies.

## Conclusions

The analysis presented here on the GAW20 data set from the GOLDN study has shown that empirical kinship is a practical alternative to pedigree-based kinships, when dense genotypic data are available, within the limitations of this study of a data set with little unknown kinship. Although we only examined phenotypes with moderate heritability, it is likely that the near functional equivalence of empirical and pedigree relatedness matrices holds across the spectrum of heritabilities. Analytical theory supports this as the expected power across heritabilities is determined by the eigenvalues of the relatedness kernel itself [16]. In this data set heritability estimates of triglyceride and HDL-C phenotypes obtained using empirical kinships from IBDLD more closely resembled those obtained with the pedigree based kinship estimations than those obtained using LDAK-based empirical kinships. The phenotypes assessed here were found to be highly and significantly heritable and measured genotype association testing identified a single variant, rs247617, as significantly associated with variation in HDL-C in line with the known biology of the gene closest to this variant, *CETP*.
